# The Primary Antisense Transcriptome of *Halobacterium salinarum* NRC-1

**DOI:** 10.3390/genes10040280

**Published:** 2019-04-05

**Authors:** João Paulo Pereira de Almeida, Ricardo Z. N. Vêncio, Alan P. R. Lorenzetti, Felipe ten-Caten, José Vicente Gomes-Filho, Tie Koide

**Affiliations:** 1 Department of Biochemistry and Immunology, Ribeirão Preto Medical School, University of São Paulo, São Paulo 14049-900, Brazil; jpereiradealmeida.mg32@gmail.com (J.P.P.d.A.); alan.lorenzetti@gmail.com (A.P.R.L.); ftencaten@gmail.com (F.t.-C.); vicente.gomes.filho@gmail.com (J.V.G.-F.); 2 Department of Computation and Mathematics, Faculdade de Filosofia, Ciências e Letras de Ribeirão Preto, University of São Paulo, São Paulo 14049-900, Brazil; rvencio@usp.br

**Keywords:** antisense RNA, *Halobacterium salinarum*, transcriptome, dRNA-seq, archaea, transcription start site, post-transcriptional regulation, gene expression, type II toxin-antitoxin systems, Ribo-seq

## Abstract

Antisense RNAs (asRNAs) are present in diverse organisms and play important roles in gene regulation. In this work, we mapped the primary antisense transcriptome in the halophilic archaeon *Halobacterium salinarum* NRC-1. By reanalyzing publicly available data, we mapped antisense transcription start sites (aTSSs) and inferred the probable 3′ ends of these transcripts. We analyzed the resulting asRNAs according to the size, location, function of genes on the opposite strand, expression levels and conservation. We show that at least 21% of the genes contain asRNAs in *H. salinarum.* Most of these asRNAs are expressed at low levels. They are located antisense to genes related to distinctive characteristics of *H. salinarum*, such as bacteriorhodopsin, gas vesicles, transposases and other important biological processes such as translation. We provide evidence to support asRNAs in type II toxin–antitoxin systems in archaea. We also analyzed public Ribosome profiling (Ribo-seq) data and found that ~10% of the asRNAs are ribosome-associated non-coding RNAs (rancRNAs), with asRNAs from transposases overrepresented. Using a comparative transcriptomics approach, we found that ~19% of the asRNAs annotated in *H. salinarum* belong to genes with an ortholog in *Haloferax volcanii*, in which an aTSS could be identified with positional equivalence. This shows that most asRNAs are not conserved between these halophilic archaea.

## 1. Introduction

Antisense RNAs (asRNAs) are non-coding RNAs (ncRNAs) transcribed from the opposite strand of a given gene. Intuitively, asRNAs can be assumed to be cis-acting as they are complementary to the messenger RNA (mRNA) of the gene from which they derive. This does not restrict asRNA action to the gene on the opposite strand, they can also act in trans [1]. AsRNAs can act as regulators at different stages of gene expression [2]. They can modulate the stability and lifespan of RNAs by either occluding degradation sites or forming double-stranded complexes (dsRNAs) that are targets for RNases [3]. Furthermore, they may directly influence translation, inhibiting this process by occluding ribosome binding sites or promoting conformational alterations that might increase or decrease the frequency of translation of its target [2,4,5].

Simultaneous transcription of opposite DNA strands was first reported in λ phage [6], later it was identified in bacteria in processes related to plasmid replication, phage repression and transposases [7], and finally in eukaryotes [8]. Technical advances in transcriptome analysis allowed greater confidence in strand-specific datasets, allowing the identification of asRNAs in different organisms [9,10,11,12]. Overlapping antisense transcription is a ubiquitous phenomenon [13,14,15], while the functionality of these molecules is still under debate, since it can arise from spurious transcription [16]. In bacteria the percentage of protein coding genes with asRNAs varies from 2% in *Salmonella* [17] to up to 80% in *Pseudomonas aeruginosa* [18]. In archaea, asRNAs have been reported for a while [19,20]. Some studies assured that they were primary transcripts: 1244 in *Haloferax volcanii* [21], 1110 in *Methanolobus psychrophilus* [22], 1018 in *Thermococcus kodakarensis* [23], 48 in *Methanosarcina mazei* Gö1 [24], 29 in *Thermococcus onnurineus* NA1 [25], and 12 in *Methanocaldococcus jannaschii* DSM [26]. In *H. volcanii*, the only halophile represented, asRNAs are present in genes related to chemotaxis, transcription regulation, and insertion sequences [27].

*Halobacterium salinarum* is an extreme halophile archaeon, thriving in 4.3 M NaCl concentrations [28]. Historically known for its bacteriorhodopsin light-dependent proton pump [29], *H. salinarum* has a wide metabolic versatility for energy production such as amino acid oxidation, anaerobic respiration using different electron acceptors, and arginine fermentation [30,31]; it also shows high tolerance to diverse environmental stresses [32,33,34]. The plethora of transcriptional data in different environmental conditions and genetic backgrounds allowed the construction of a global gene regulatory network based primarily on mRNA levels [35,36]. Many overlapping features in *H. salinarum* genome, such as alternative promoter usage in operons [37] and internal RNAs that produce protein isoforms [38], have been reported, highlighting the complexity of transcriptional regulation. In addition, ncRNAs in intergenic regions [37], RNAs associated to transcription start sites (TSSaRNAs) [39], and associated to insertion sequences (sotRNAs) [40] have also been identified. However, there is little information on antisense RNAs. The first asRNA reported in this organism was a 151 nucleotide (nt) molecule complementary to the 5′ end of a phage related gene [41,42]; asRNAs regulating gas vesicles genes were also identified [19], but no further global analysis of asRNAs have been performed since then.

In this work, we report a genome-wide primary asRNA mapping in *H. salinarum* NRC-1. To do that, we took advantage of published differential RNA-sequencing (dRNA-seq) data for the identification of transcription start sites antisense to annotated genes (aTSS) [38]. To annotate the minimum length of asRNAs, we reanalyzed published strand-specific RNA-seq data acquired in paired-end mode. Data was manually inspected to guide asRNA annotation of probable 3′ ends. Publicly available Ribosome profiling (Ribo-seq) data (Raman et al. submitted) were reanalyzed to identify asRNAs bound to ribosomes. We investigated asRNA global properties: size distribution, location relative to the cognate genes, expression levels, and cognate gene functions. Finally, we did a comparison with dRNA-seq results of *H. volcanii* to evaluate the cross-species conservation of asRNAs.

## 2. Materials and Methods

### 2.1. Antisense Transcription Start Sites Annotation

We reanalyzed publicly available raw dRNA-seq data from *H. salinarum* NRC-1 grown in complex media (250 g/L NaCl, 20 g/L MgSO_4_, 2 g/L KCl, 3 g/L sodium citrate, and 10 g/L bacteriological peptone (Oxoid)) over a growth curve sampled at 17 h, 37 h, and 86 h, and grown under standard conditions (37 °C, 225 rpm, constant light) sampled at mid-log phase [38]. We used our in-house workflow (“Caloi-seq”, https://github.com/alanlorenzetti/frtc/), described in [38], for *H. salinarum* dRNA-seq data. Briefly, libraries were downloaded from NCBI Sequence Read Archive (SRA) [43] and trimmed using Trimmomatic [44]. Reads surviving as a pair were aligned to reference genome (NCBI Assembly ASM680v1) in paired-end mode using HISAT2 [45], suppressing alignments resulting in fragments longer than 1000 nt. Orphan R1 and R2 sequences were aligned using the single-end mode. Multimappers aligning up to 1000 times were allowed to be reported. SAM files were converted to BAM using SAMtools [46] and input in MMR to find the most likely position for each multimapper [47]. The resultant BAM files were filtered to keep only R1 reads, which serve as input for the TSS inference step. BEDTools [48] was used to compute the fragment 5′ accumulation profile, employing all the aligned R1 reads. Data visualization was performed using IGV [49] and Gaggle Genome Browser [50].

TSSs were identified from dRNA-Seq experiments using TSSAR [51] with the following parameters: *p*-value *p* < 0.005, a minimum of four reads, and a distance of TSS grouping of at least five nt. An antisense TSS (aTSS) was defined based on genome annotation of *H. salinarum* NRC-1 available at RefSeq updated in 2017 with additional manual curation using *H. salinarum* R1 annotation as reference [52] (Table S1). TSSs located inside genes on the opposite strand were classified as aTSS. TSSs on the opposite strand and up to 200 nt downstream of a gene 3′ end were considered downstream aTSS (daTSS).

Potentially structured regions were filtered out by calculating folding minimum free energy (MFE) along the whole genome sequence as previously described [38]. A sliding window (51 nt) with an offset of 10 nt was used to tile the genome and all subsequences were subjected to secondary structure prediction using RNAfold [53] with default parameters. The distribution of MFE obtained for the tiled genome was compared with the distribution obtained for only subsequences immediately downstream of aTSS. The 33.3% quantile in the whole-genome MFE distribution was arbitrarily chosen as cutoff for potentially forming structures and thus putative false positive aTSS.

### 2.2. Antisense RNA Loci Inference

To annotate asRNA loci we used aTSS positions together with all TEX- strand-specific paired-end RNA-seq libraries publicly available for *H. salinarum* NRC-1. Library accessions and number of sequenced, trimmed, and aligned reads used for asRNA loci annotation are shown in Table S2. These libraries are the control libraries for a dRNA-seq experiment, representing four replicates of *H. salinarum* NRC-1 grown under standard conditions and sampled at mid-log phase and biological duplicates of three different time points over a growth curve (17 h, 37 h, and 86 h) [38]. For these libraries, we took the MMR adjusted BAM files, cited in the last section, and computed the genome-wide coverage (transcriptional profile) using deepTools [54], considering the extension of full fragments for paired-end alignments and the proper strand orientation for alignments of R1 and R2 orphan reads. BEDTools [48] was used to compute the fragment 3ʹ accumulation profile, employing all the aligned R2 reads. Visual inspection was performed using IGV [49] and Gaggle Genome Browser [50] by looking for a mapped aTSS followed by a sharp decrease in read coverage to infer an asRNA locus in at least four different libraries. To aid the inference of locus ending, we used the fragment 3′ end accumulation profile, requiring at least 10 observations to demarcate the minimum end position of an asRNA. The steps for processing dRNA-seq data and computing the transcriptional profiles are depicted as a workflow in Figure S1, and asRNA loci inference is schematically shown in Figure S2.

### 2.3. Promoter and 3′ End Sequence Analysis

The computation of nucleotide frequency for the promoter region of aTSSs and 3′ end of asRNAs was performed using Weblogo [55]. For the promoter region we analyzed sequences 40 nt upstream and 10 nt downstream of the aTSS; for the 3′ end, we used sequences 10 nt upstream and downstream of the last nucleotide.

### 2.4. Gene Functions

Gene functional classification and Gene Ontology enrichment analysis were performed using the PANTHER system [56]. In the absence of an associated PANTHER annotation, MicrobesOnLine [57] and BlastCD [58] tools were used to identify conserved protein domains.

### 2.5. Type II Toxin-Antitoxin Systems Annotation and Antisense Transcription Start Sites Identification in Other Archaea

TA finder 2.0 [59] was used to annotate type II Toxin-Antitoxin (TA) systems in *E. coli* and archaeal organisms with available dRNA-seq data. aTSS positions were mapped as described in Section 2.1, reanalyzing dRNA-seq data from NCBI BioProject database for *Haloferax volcanii* DS2 (PRJNA324298), *Methanocaldococcus jannaschii* DSM 2661 (PRJNA342613), *Thermococcus kodakarensis* KOD1 (PRJNA242777), and *Thermococcus onnurineus* NA1 (PRJNA339284). aTSS positions for *E. coli* were obtained from Thomason et al. [60], and positions of asRNAs in type II TA systems found in an immunoprecipitation ofdsRNAs experiment were obtained from Lybecker et al. [61].

### 2.6. Differential Expression Analysis

A GFF file including *H. salinarum* NRC-1 annotated genes, in addition to the 846 asRNAs identified in this study, was built, generating a total of 3680 features. Matrices for read counts per feature were generated using HT-Seq [62] from BAM files. Differential expression analysis was performed using DEseq2 [63]. Genes with more than two-fold change (FC) up or down regulation (log_2_ FC > 1 or < −1) and adjusted *p*-values *p_adj_* < 0.01 were considered differentially expressed.

### 2.7. Antisense Transcription Start Sites Comparison between Halobacterium salinarum and Haloferax volcanii

Ortholog genes in *H. salinarum* and *H. volcanii* were identified using OrtholugeDB 2.1 [64]. Pairs of genes with aTSSs were sub-selected and gene sizes were normalized by their length in a 0 to 100 normalized scale (*d* = 0 at start codon, *d* = 100 at stop codon). The length-normalized genes were divided in 10 partitions (tenths) depending on *d*: [0;10), [10;20), …, [90;100), and aTSSs were considered conserved if present in the same partition in the orthologous genes. Differences between relative positions, *D* = |*d_Hsal_* − *d_Hvol_*|, were used to estimate how “equivalent” aTSS are between both organisms.

### 2.8. Ribo-Seq Data Analysis

*H. salinarum* NRC-1 Ribo-seq data is publicly available ahead of publication (Raman et al. submitted) at NCBI BioProject database under accession PRJNA413990 and was reanalyzed using the same pipeline described in Section 2.1 and Section 2.2. AsRNAs presenting coverage greater than 50% and at least 20 reads were considered putative ribosome-associated ncRNAs (rancRNAs).

## 3. Results

### 3.1. Mapping Primary Antisense RNAs

**Figure 1:**
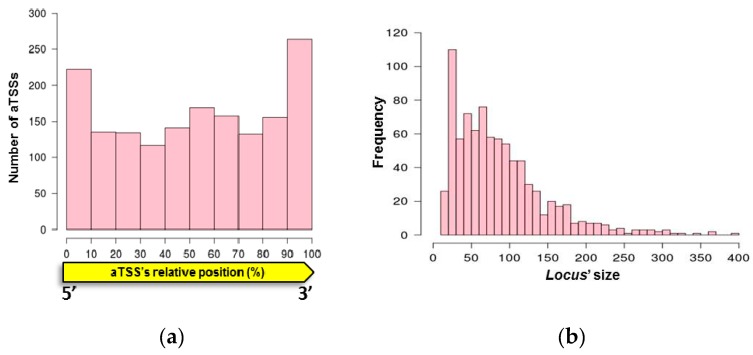
Antisense RNA (asRNA) properties. (**a**) Antisense transcription start site (aTSS) positions relative to cognate genes. (**b**) Size distribution of mapped asRNAs.

We identified the primary transcription start sites (TSS) for asRNAs (aTSS) in *H. salinarum* NRC-1 by reanalyzing dRNA-seq data sampled at three time points over growth [38]. We were able to identify 2146 aTSS, located in 1231 genes. Probable false positives due to secondary structure were filtered out (see Methods 2.1), resulting in 1626 aTSSs located in 963 genes (Table S3). Figure 1a shows the position of aTSS in a length-normalized scale (0 to 100 scale) relative to the gene on the opposite strand. The overrepresentation of aTSS at the 3′ end is stronger than at the 5′ end.

Given that (i) asRNAs can be located in regions downstream of the cognate gene, (ii) 3′ untranslated regions (UTR) can be targeted by ncRNAs, and (iii) asRNAs from 3′ UTRs are important regulatory RNAs in archaea with short 5′ UTRs [65], we searched for downstream antisense TSS (daTSS). They were defined as transcript start sites located on the opposite strand of annotated genes, located up to 200 nt downstream of the 3′ end of a gene. We found 80 daTSS (Table S4).

Having strand-specific paired-end data available, we could search for genome positions at which antisense transcripts preferentially terminate. Such presumed RNA 3′ ends, if downstream of an aTSS (RNA 5′ end), were inferred to represent the end of an asRNA locus (see Methods 2.2). We were able to define 846 asRNAs loci distributed in 613 genes, starting from aTSS or daTSS (Table S4). Most of the genes contain only one asRNA locus and 26 of the asRNAs are antisense to two genes. The size distribution of asRNAs is shown in Figure 1b, most of them being smaller than 100 nt.

We analyzed the frequency of nucleotides around aTSSs and daTSSs (Figure S3a) and observed the predominance of purines at the TSS, pyrimidines at position −1 and a BRE-TATA signature, showing a characteristic archaeal promoter region for the mapped asRNAs. The dinucleotide composition around the TSS is postulated as important for the transcription initiation among organisms from all domains [66,67,68], as well as the GC enrichment around the −36 position for TFIIB binding and TATA-box at −26 [69,70]. As previously shown, dRNA-seq data reliably identifies TSS genome-wide in *H. salinarum* NRC-1 [38], recapitulating known features of the promoter region. Nevertheless, this does not exclude the possibility that many of the asRNAs identified are products of spurious transcription.

For asRNAs with at least 10 fragments that corroborated the 3′ end annotation, we analyzed the frequency of nucleotides 10 nt upstream and downstream of the 3′ end. We observed an enrichment of pyrimidines, specially uracil at positions 0 and −1 (Figure S3b). Comparing this signature with poly-U signatures found in other archaea [71], our data shows a much shorter region, which is unlikely to form a secondary structure involved in transcription termination of asRNAs.

**Figure 2:**
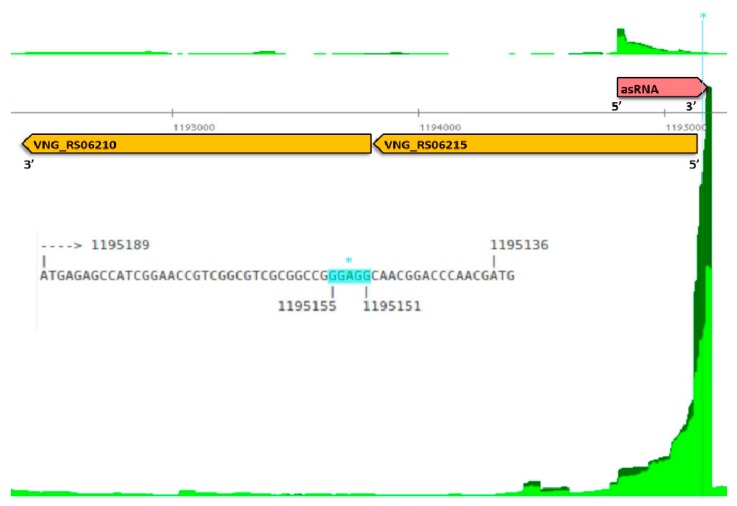
Antisense RNA in 5′ untranslated region (UTR). *gcvP1*, encoding a glycine dehydrogenase subunit (VNG_RS06215). VNG_RS06215 locus (orange arrow) is in reverse strand (5′→3′ right to left), neighbor gene VNG_RS06210 (orange arrow) is also in reverse strand. Differential RNA-sequencing (dRNA-seq) read coverage signal is shown in dark and light green for TEX+ and TEX− libraries, respectively. Coverage signals below and above the central axis are for reverse and forward strands, respectively. VNG_as06215_888 asRNA (pink arrow) encompasses Shine–Dalgarno-like signature (* light blue highlight in genome coordinates and zoomed in sequence).

Most of the characterized sRNAs in prokaryotes bind to the 5′ region of the mRNA, pairing with the start codon or ribosome binding site (RBS) in the 5′ UTR [17], and thus inhibiting the access of the ribosomal machinery. asRNAs that overlap these regions can be candidates to act through a similar mechanism. Halophiles such as *H. salinarum* NRC-1 and *H. volcanii* have mostly leaderless transcripts [21,37,72]. However, it has been experimentally shown in *Salmonella* that ncRNAs overlapping nucleotides up to the 5th codon in the mRNA are capable of blocking the translation machinery, even without AUG or Shine–Dalgarno pairing [73]. We thus looked for asRNAs that overlap their cognate gene′s start codon or at least 12 nt (four codons) downstream of it. We found 145 asRNAs in *H. salinarum* overlapping the 5′ end of genes (Table S5), which could, in principle, impair mRNA translation by occluding ribosome binding. Figure 2 and Figure S4 show examples of putative RBS occlusion by an asRNA: *gcvP1*, encoding a glycine dehydrogenase subunit (VNG_RS06215); *cdcH*, encoding an AAA-type ATPase (VNG_RS06465); and *rpl1*, which encodes the 50S ribosomal protein L1 (VNG_RS04315). The gene *gcvP1* is the first from an operon and has a strong Shine–Dalgarno-like signature at −19 upstream of the start codon, colocalized with asRNA VNG_as06215_888 (Figure 2). Similarly, RBS at −11 and −13 upstream of *rpl1* and *cdcH* are colocalized with asRNA VNG_as04315_654 and VNG_as06465_925, respectively (Figure S4).

All data are available for browsing in Gaggle Genome Browser format (interactive versions of Figure 2 and similar outputs) at http://labpib.fmrp.usp.br/~rvencio/asrna/.

### 3.2. Antisense RNAs Expression Levels

Some of the asRNAs with characterized functions are expressed at levels equivalent or higher than the mRNA on the opposite strand, which would be expected if a dsRNA is necessary for post-transcriptional regulation [74]. However, most of the asRNAs identified in eukaryotes and prokaryotes are expressed at low levels, which has challenged their identification before high-resolution sequencing methods were available [2,16,60]. Low expression levels might indicate that these asRNAs are products of spurious transcription from low complexity promoter regions [16]. However, this does not exclude the possibility of a functional RNA, given that even at low levels, asRNAs can present a buffering effect and fine-tune gene regulation [2,3].

**Figure 3:**
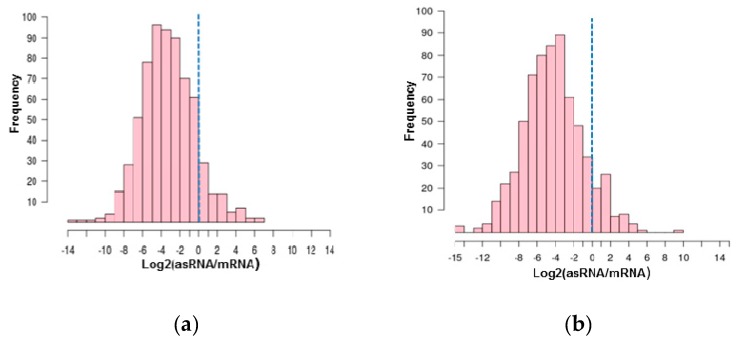
Relative expression levels of asRNAs and messenger RNA (mRNAs) on the opposite strand for arbitrarily selected representative libraries: (**a**) stationary phase (17 h) and (**b**) gas vesicle release phase (86 h). Vertical dotted lines mark 1:1 expression levels. Expanded version in Figure S5.

We observe a negative correlation between asRNA and mRNA transcripts when we compare the average read counts for these transcripts (Figure S5a), as observed in *H. volcanii* [21]. Using RNA-seq data, we analyzed the relationship between the fold change of the read counts of asRNAs relative to the mRNA on the opposite strand (Figure 3). We observed that most of the asRNAs annotated in our study present low expression levels relative to the gene on the opposite strand. Only 112 asRNAs (~13%, Table S6) present expression levels equal or greater than the gene on the opposite strand in at least one of the conditions analyzed. These molecules could be candidates for *cis*-regulators of their respective cognate genes in the considered experimental conditions.

We also evaluated the expression profiles of the asRNAs and cognate genes over the growth curve of *H. salinarum* NRC-1 using RNA-seq libraries sampled at three time points [38]. We compared the expression levels from (i) the stationary phase relative to exponential phase (17 h vs. 37 h) and (ii) late exponential gas vesicle release phase relative to stationary phase (37 h vs. 86 h).

In the first transition, we observed 93 asRNA differentially expressed (Table S7). For 26 asRNAs, the gene on the opposite strand is also differentially expressed (Figure S6a), and six asRNAs overlap the 5′ UTR of the genes. In the second transition, we observed 63 asRNAs differentially expressed (Table S8). For 30 cases, the gene on the opposite strand is also differentially expressed. Twenty-seven pairs were differentially expressed in the same direction and only three in opposite directions (Figure S6b). Of them, seven asRNAs overlap the 5′ UTR region. Overall, 56 pairs of gene-asRNAs are differentially expressed in at least one of the transitions analyzed. These asRNAs can be potential *cis* regulators of the cognate genes.

### 3.3. Function of the Genes on the Opposite Strand of Antisense RNAs

From the total of 613 genes that present at least one asRNA locus, 198 are hypothetical proteins and 32 are nonredundant transposases (i.e., some of them are multicopy, but counted only once). Gene enrichment analysis returned no overrepresented Gene Ontology (GO) term relative to the whole genome background distribution. Genes with asRNAs are associated with different functions according to GO categorization (Figure S7). Next, we present the results for some of the gene categories.

*H. salinarum* NRC-1 is known to produce intracellular gas vesicles: structures composed of proteins that are filled with gas and allow floating on the media surface. Their synthesis and regulation have been studied in details and includes multiple layers of regulation [75]. We were able to identify 11 asRNAs in the *gvp* gene cluster (Table 1), including one previously identified on the opposite strand of *gvpD* [19], which encodes a repressor of gas vesicle production. Most asRNAs related to *gvp* genes showed similar expression levels relative to their cognate genes.

AsRNAs to the *gvpD* gene have been detected by Krüger and Pfeifer [19], complementary to the 5′ and 3′ end of the gene and detected when GvpD protein was present at low levels. We were able to identify three RNAs antisense to *gvpD*, one of them (VNG_as12315_1568) overlaps the 5′ end that recapitulates previously published information. This asRNA was annotated as a 164 nt molecule in our work, presenting a similar size to the 190 nt band observed in [19]. The aTSS mapped is located 3 nt downstream to the 5′ end of the probe used in the previous work, showing the reliability of our asRNA mapping (Figure S8). We also identified primary asRNAs colocalized with genes *gvpC*, *gvpH*, *gvpI*, *gvpJ*, *gvpL*, and a strong signal antisense to the 5′ end of *gvpA*, although no aTSS was mapped (Figure S8). These data indicate asRNAs as important players in gas vesicle regulation in *H. salinarum*, as reported in cyanobacteria *Calothrix* sp. PCC 7601 [76].

Moreover, we were able to identify RNAs possibly involved in rhodopsin regulation in *H. salinarum*: asRNAs in *bop* (VNG_RS05715—bacteriorhodopsin) and its regulators *brz* (VNG_RS05710—bacteriorhodopsin regulating zinc finger protein) and *brb* (OE3105F bacteriorhodopsin regulating basic protein), in addition to an asRNA in halorhodopsin (VNG_RS00745) (Table 2). Brb fine-tunes the activation of *bop*; this activity was experimentally shown using reporter genes and mutagenesis. The Brb protein was proven to exist, but was not detected using mass spectrometry [77]. The asRNA overlapping bacteriorhodopsin regulators (VNG_da3105F_36) starts downstream of *brb*, overlaps it completely, and ends inside *brz* gene (Figure S9). In the RNA-seq libraries analyzed in this study, the number of reads for this asRNA was approximately 4-fold higher than for *brb* gene. If asRNAs should be present at high levels to post-transcriptionally regulate an mRNA, this data could account for the difficulty in Brb protein detection since asRNA–mRNA pairing could block translation, indicating a possible role for this asRNA. The presence of asRNAs in bacteriorhodopsin and halorhodopsin genes could indicate additional regulators to be studied for understanding the photobiology of this organism.

In bacteria, type I TA systems are known for the presence of an asRNA acting as an antitoxin, while in type II TA systems, both toxin and antitoxin are known to be proteins [78]. In 2014, Lybecker et al. [61] found asRNAs to type II TA systems in *E. coli*, indicating a possible role for asRNAs in these systems. In *H. salinarum* NRC-1, we identified asRNAs to genes VNG_RS11240 and VNG_RS00140, which are annotated type II antitoxins.

The presence of asRNAs in type II TA systems has not been systematically explored and could indicate another layer of regulation for these systems. To verify the compatibility of TSS identification and asRNAs identified by Lybecker et al. using dsRNA immunoprecipitation [61], we reanalyzed dRNA-seq data from *E. coli* [60], and we were able to detect the aTSS corresponding to the asRNAs reported by Lybecker et al. [61] in type II TA systems. Given that aTSS identification was reliable in *E. coli*, we looked for asRNAs in type II TA systems in archaea by precisely annotating these genes using TA finder 2.0 [59] and reanalyzing available dRNA-seq data for *H. salinarum* NRC-1 (PRJNA448992) [38], *H. volcanii* DS2 (PRJNA324298) [21], *M. jannaschii* DSM 2661 (PRJNA342613) [26], *T. kodakarensis* KOD1 (PRJNA242777) [23], and *T. onnurineus* NA1 (PRJNA339284) [25]. We annotated new type II TA systems in archaea (Table S9), including nine complete pairs in *H. salinarum*. Both genes composing one of these new pairs, VNG_RS11890 (toxin) and VNG_RS11895 (antitoxin), have asRNAs displaying expression levels higher than the cognate genes (Figure S10). Most of the annotated type II TA systems have at least one aTSS in one of the genes. For *T. kodakarensis* and *M. jannaschii*, aTSSs are predominantly in the toxin genes.

We found asRNAs for 37 genes related to translation process, including ribosomal proteins, translation initiation factors, transfer RNA (tRNA) ligases and tRNAs, Asn, Lys, and Ser (Table S10). There are reports of RNAs antisense to tRNAs in *S. solfataricus* [79] and *T. kodakarensis* [23], which indicate a conserved regulatory role for these molecules.

Interestingly, 58 out of 846 asRNAs are overlapping transposase genes in *H. salinarum* NRC-1 (Table S11). Since transposases are usually encoded within repetitive elements called insertion sequences (IS), we eliminated redundancy in numbers by choosing only one representative element for multicopy entities. AsRNAs in transposases have been reported in other archaea such as *H. volcanii*, *T. kodakarensis*, *S. solfataricus*, and *M. mazei* [20]. Retrieving legacy data from tiling microarray experiments performed along *H. salinarum* growth curve [37], we verified that several transcripts antisense to IS are differentially expressed (Figure S11).

In bacteria, there are examples of asRNA inhibiting the translation of transposase mRNAs by occluding the ribosomal machinery assembly at the 5′ end of an mRNA [80]. We found 10 asRNAs that overlap the 5′ end of transposase coding gene (highlighted in Table S11), which could be potential candidates for a similar regulatory mechanism.

### 3.4. Ribosome-Associated Antisense RNAs

We reanalyzed Ribo-seq data, (BioProject PRJNA413990), to identify asRNAs that are putative ribosome-associated ncRNAs (rancRNAs) [81]. By looking at asRNA loci covered by at least 20 reads along at least half its extension, we identified 91 asRNAs (~11%) with relevant signal (Table S12). Recently published rancRNA data in *H. volcanii* found 68 candidates antisense to genes, ~6% of their total [82].

Interestingly, 11 asRNAs with relevant Ribo-seq signal are located in transposases (representative instances highlighted in Table S12). This might indicate that these asRNAs can be either targets for translation or regulate/interfere with the ribosomal machinery. Given that years of *H. salinarum* proteomics studies refuted a widespread colocalization of open reading frames in both strands as spurious “overprediction” [52], the regulatory ribosome binding scenario seems more plausible.

### 3.5. Conservation of Antisense Transcription Start Sites 

Transcriptome analysis of bacteria has shown that the conservation of asRNAs even among phylogenetically close organisms is low. The comparison between *E. coli* and *Salmonella enterica* serovar Typhimurium showed that only 14% of the asRNAs are conserved [83]. The number of conserved aTSSs varies in different organisms: comparison between *Campylobacter* strains showed 45% conservation [84]; within eight different species of *Shewanella* genus, 22% [85]; and among *Synechocystis* strains, only 4% [86].

**Figure 4:**
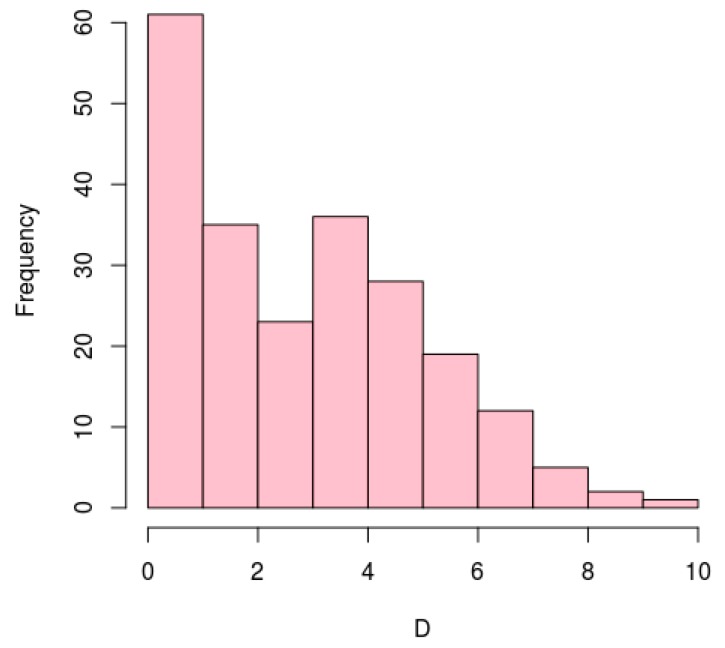
Distribution of differences (*D*) between relative positions among aTSS conserved in *H. salinarum* NRC-1 and *H. volcanii* DS2. Positions are coding sequences (CDS) length-normalized thus *D* = 0 mean same position in both organisms.

To evaluate the conservation of aTSS, we compared the identified positions in *H. salinarum* with dRNA-seq data reanalysis of *H. volcanii.* First we sub-selected pairs of orthologous genes in both halophiles (1554 pairs) [64]. Then, from these groups of orthologous genes, we sub-selected pairs with an annotated asRNA in *H. salinarum*′s genes and at least one aTSS in its correspondent ortholog in *H. volcanii*, obtaining 244 pairs. We normalized genes sizes by coding sequences (CDS) length defining *d* = 0 at start codons and *d* = 100 at stop codons. We arbitrarily partitioned CDS in 10 regions depending on *d*: (0;10), (10;20), and so on up to (90;100). Then, we searched for annotated asRNAs in *H. salinarum*’s genes and aTSSs in their correspondent ortholog in *H. volcanii* located in the same “equivalent” region. We were able to identify 160 asRNAs, distributed in 110 *H. salinarum*’s genes that contain at least one aTSS in the same tenth region of its ortholog gene in *H. volcanii* (Table S13), representing ~19% of the annotated *H. salinarum* asRNAs. We deemed an aTSS as a conserved feature if its relative position falls into the same tenth partition in both organisms. The distribution of differences between relative positions (*D* = |*d_Hsal_* − *d_Hvol_*|) shows that the majority of such 160 conserved aTSS are in fact positionally equivalent (*D* < 3) (Figure 4).

We found only seven genes in which aTSS are found exactly at the same relative position (*D* = 0): *aglM2*, *rpl10e*, *eEF1A*, *rpoB1*, *ndhG4*, *nirH*, and *atpI.* Two of them are also equal in absolute positions since they have the same length in both species (highlighted in Table S13).

Currently, *H. volcanii* is the only other halophilic archaeon for which primary transcriptome is available (dRNA-seq data). Regular strand-specific comparative transcriptome analysis (RNA-seq) is available for other halophiles but only one, in *Natrinema* sp. J7-2 (formerly *H. salinarum* J7), focused on salinity adaptation questions [87]. Since regular RNA-seq does not have the resolution power to pinpoint aTSS as dRNA-seq, we inspected differential read distributions between *Natrinema* sp. J7-2 cultures grown on relatively low vs. high salt concentrations (15% vs. 30% NaCl) guided by the *H. salinarum* and *H. volcanii* conservation results. From the seven aforementioned genes bearing conserved aTSS, only *nirH* (which encodes a sirohaem decarboxylase) is clearly upregulated in low salt relative to high salt, while its putative conserved asRNA is upregulated in high salt relative to low salt concentrations (Figure S12).

Recently published oxidative stress data in *H. volcanii* [27] was investigated to find an intersection between differentially expressed asRNAs in *H. salinarum* NRC-1. Among the 160 conserved asRNAs, four and 15 were down- and upregulated in the *H. volcanii* oxidative stress response (Table S14). From these, two were also found differentially expressed in 37 h/17 h transition: antisense to *rmeS*, encoding restriction endonuclease subunit (VNG_RS00420), and *pyrG*, encoding a CTP synthase (VNG_RS07090). Analogously, three were found in the 86 h/37 h transition: antisense to a ISH8-type transposase (VNG_RS00205); *lon*, encoding an archaeal Lon protease (VNG_RS01200); and *csg*, encoding the S-layer glycoprotein of *H. salinarum* (VNG_RS10505).

## 4. Discussion

In this study, we were able to define a map of the primary antisense transcriptome in *H. salinarum* NRC-1, corroborating the pervasiveness of antisense transcripts in this organism. The first step was to reanalyze dRNA-seq data, where RNAs with triphosphorylated 5′ ends were enriched by treatment with terminator exonuclease and then compared to untreated samples. It was possible to precisely identify aTSSs, which were further filtered to remove possible artifacts due to secondary structure resulting in 1626 aTSSs. We could observe a typical archaeal promoter structure upstream of the mapped aTSSs (Figure S3a), showing that we can confidently identify these positions. In *H. volcanii*, it was reported that ~30% of the genes present an aTSS during exponential growth [21], in *H. salinarum* we observed similar figure of ~34%. We also observed a similar distribution of aTSS relative to the sense gene with an accumulation at the 3′ end (Figure 1a), suggesting that in halophiles aTSS are preferentially located at this region.

Even with high resolution genomic techniques, the definition of the precise 3′ end of a transcript can be challenging since the signal decrease is usually not very sharp and termination signatures are still being discovered, especially in archaea [71]. In the studies using dRNA-seq to analyze the primary transcriptome of *E. coli* [60] and *H. volcanii* [21], the size of the asRNAs were arbitrarily defined as 50 and 100 nt, respectively. Visual inspection of the RNA-seq signal has been used as a valid approach to infer the 3′ end of diverse ncRNAs in bacteria and archaea [27,67,88,89]. In this work, we not only used the visual inspection of 10 RNA-seq libraries, but also the information of paired-end sequencing, which is routinely used to map transcriptomes and genomes in eukaryotes [90]. This approach allowed us to increase the confidence in the inference of the possible 3′ end, defining the minimum size of the asRNA. We observed a short uracil enrichment at the two last positions of the asRNAs (Figure S3b), which might indicate some sort of termination signature, although it is too short to form a termination structure as previously reported in Santangelo and Reeve [91] and Dar et al. [71]. The application of different library preparation methods such as transcription termini sequence (Term-seq) or long reads sequencing [71,92] can help infer the exact size of a transcript to study their properties in the future.

We defined 846 asRNA loci with an aTSS and a 3′ end that marks their minimum length; this is probably the minimum list of asRNA primary transcriptome of *H. salinarum*. There are several other antisense signals, such as aTSS, for which we were not able to define a 3′ end, long 3′ UTR regions of mRNAs, or RNAs that are not primary (possibly processed). An example is shown in Figure S13, where a long 3′ UTR of a coding sequence can act as an asRNA for a translation initiation factor and Figure S8 where a strong signal antisense to *gvpA* is detected, although no TSS could be mapped. Most of the characterized sRNAs in prokaryotes bind to the 5′ UTR of mRNA, inhibiting ribosome binding, and thus blocking translation [17]. However, in *H. salinarum* most of the transcripts are leaderless, which could account for the overrepresentation of asRNAs overlapping 3′ ends of mRNAs (Figure 1a). Nevertheless, we identified 145 asRNAs that could impair translation by overlapping 5′ end region of the mRNA.

Although unquestionably prevalent, asRNAs are usually expressed at low levels, which brings to discussion whether these molecules are functional or by-products of a noisy transcription process. If an asRNA has to pair with an mRNA and form a dsRNA to perform a post-transcriptional regulation, it would be expected that, given stoichiometric considerations, asRNAs would be present at similar levels of the target RNAs [74]. In our study, only 112 (~13%) of the asRNAs present expression equal to or greater than the cognate gene. Many bacterial asRNAs can be spurious transcripts that would be maintained in the genome due to the absence of a negative selection (low energy cost or no deleterious effects) [16]. However, low expression levels do not exclude the possibility of a functional molecule, given that they could interact with different proteins or present a buffering effect to fine-tune mRNA regulation [2]. Even if expressed at low levels and possibly non-functional, many of the asRNAs are detected in different conditions by transcriptomics methods, indicating that they could be a source for evolutionary processes that originate new regulatory elements, as molecular exaptations [93].

Concerning the function of genes with an asRNA, we could find many interesting examples that might present antisense regulation. We were able to recapitulate previously reported information on asRNA to *gvpD* gene, adding the information that this is a primary transcript. Another 10 asRNAs in the *gvp* gene cluster were identified, suggesting a post-transcriptional regulation of these genes yet to be described. In genes related to bacteriorhodopsin, we found a long asRNA that, if it hybridizes with *brz* and *brb* mRNAs, could block their translation, which could explain the difficulty in experimentally detecting Brb protein in physiological conditions [77]. Type II TA systems were found to present aTSS not only in *H. salinarum* but also in three other archaea with available dRNA-seq data, suggesting a conserved role of asRNAs in type II TA systems. AsRNAs were also found in 37 genes related to translation and 32 transposases. For this last group 31% has a clear overlap with the 5′ end, suggesting the mechanism of translation inhibition of these genes. We found that ~11% of the asRNAs interact with ribosomes. This could indicate that these asRNAs are either encoding unknown proteins or regulating ribosomal machinery in yet to be described ways.

Expression levels of asRNA–mRNA pairs were evaluated over a growth curve, and we found that 17% of annotated asRNAs show either positive or negative correlation with the expression of the cognate gene. Among these pairs, we found a toxin from a type II TA system (VNG_RS00140) upregulated while the asRNA (VNG_da00140_3) is downregulated (Figure S6a). This negative correlation is usually found in type I TA systems [4], where the asRNA is an antitoxin. We found 27 hypothetical proteins differentially expressed with their asRNA also differentially expressed. In *H. volcanii*, differential expression during oxidative stress identified 48% of the genes that encoded hypothetical proteins, along with their respective asRNAs [27]. This suggests a probable regulation of these genes by asRNAs in halophiles, although the impact of these genes is still unknown. Although differential expression analysis is commonly performed when studying asRNAs, it is important to note that this is a simplistic approach, since sense and antisense gene regulation might be completely independent [94] and mRNA levels are not necessarily correlated to protein levels [95]. This list of differentially expressed asRNAs–mRNAs can be a first approach to select targets for further experimental validation in *H. salinarum*.

Conservation of asRNAs is usually reported as very low, even among closely related organisms. We found that only ~19% of the aTSSs with asRNAs annotated are conserved between *H. salinarum* and *H. volcanii*, with two examples where they are found at exactly the same location. To infer the functionality of a given gene, a premise is that along the evolutionary process the sequence will be conserved between species. The low conservation of asRNAs, as well as their low expression levels, raise the question about the functionality of most asRNAs identified by high-throughput methods [85]. Higher evolution rates in ncRNAs could be related to a faster adaptation of organisms to remodel regulatory pathways to generate specific responses, which could be applied to the asRNAs [2,86].

## Figures and Tables

**Table 1:** asRNAs in *gvp* gene cluster, located in pNRC100. (nt: Nucleotide).

aTSS ID	asRNA ID	Strand	Start	End	Size (nt)	Locus	Annotation
aTSS_1555	VNG_as12280_1555	+	16743	16863	120	VNG_RS12280	*gvpL*
aTSS_1556	VNG_as12280_1556	+	17055	17138	83	VNG_RS12280	*gvpL*
aTSS_1557	VNG_as12290_1557	+	18092	18168	76	VNG_RS12290	*gvpJ*
aTSS_1558	VNG_as13760_1558	+	18390	18428	38	VNG_RS13760	*gvpI*
aTSS_1559	VNG_as13760_1559	+	18615	18643	28	VNG_RS13760	*gvpI*
aTSS_1560	VNG_as12295_1560	+	18698	18722	24	VNG_RS12295	*gvpH*
aTSS_1565	VNG_as12315_1565	+	21000	21165	165	VNG_RS12315	*gvpD*
aTSS_1567	VNG_as12315_1567	+	22084	22106	22	VNG_RS12315	*gvpD*
aTSS_1568	VNG_as12315_1568	+	22128	22292	164	VNG_RS12315	*gvpD*
aTSS_1569	VNG_as12325_1569	−	22865	22964	99	VNG_RS12325	*gvpC*
aTSS_1570	VNG_as12325_1570	−	23888	23940	52	VNG_RS12325	*gvpC*

**Table 2:** asRNAs in rhodopsin related genes, located in the main chromosome.

aTSS ID	asRNA ID	Strand	Start	End	Size (nt)	Locus	Annotation
aTSS_175	VNG_as00745_175	+	155806	155906	100	VNG_RS00745	*halorhodopsin*
*daTSS_36	VNG_da3105F_36	−	1088797	1089100	303	VNG_RS05710 VNG_OE3105F	*brz—bacteriorhodopsin regulating zinc finger protein;* *brb—bacteriorhodopsin-regulating basic protein*
aTSS_824	VNG_as05715_824	−	1089545	1089615	70	VNG_RS05715	*bacteriorhodopsin*

* The asRNA VNG_da3105F_36 overlaps two genes.

## Supplementary Materials

The following are available online at https://www.mdpi.com/2073-4425/10/4/280/s1: Figure S1: Pipeline for asRNA annotation, Figure S2: asRNA loci inference approach, Figure S3: asRNA properties, Figure S4: Examples of putative RBS occlusion by an asRNA, Figure S5: Coverage of asRNAs relative to mRNAs on the opposite strand, Figure S6: Differential expression of asRNAs and mRNAs on the opposite strand, Figure S7: Functional categorization of genes presenting asRNAs according to Gene Ontology (GO), Figure S8: asRNAs in *gvp* genes, Figure S9: asRNAs in *brb* and *brz* genes, Figure S10: asRNAs in type II TA system, Figure S11: asRNAs antisense to a transposase differentially expressed along the growth curve, Figure S12: Differential expression of putative asRNAs antisense to the *nirH* gene in *Natrinema* sp. J7-2, Figure S13: Long 3′ UTR of a coding sequence acting as an asRNA, Table S1: Genome annotation of *H. salinarum* NRC-1 (RefSeq updated in 2017) with additional manual curation using *H. salinarum* R1 annotation as reference, Table S2: RNA-seq data used to annotate asRNA loci, Table S3: aTSS mapped in *H. salinarum* NRC-1, Table S4: asRNA loci mapped in *H. salinarum* NRC-1, Table S5: asRNAs overlapping 5′ end of genes, Table S6: asRNAs with expression levels equal or greater than the gene on the opposite strand, Table S7: asRNAs differentially expressed in the stationary phase relative to exponential phase (17 h vs 37 h), Table S8: asRNAs differentially expressed in the late exponential gas vesicle release phase relative to stationary phase (37 h vs 86 h), Table S9: Annotation and aTSSs in type II TA systems in archaea, Table S10: asRNAs in genes related to translation, Table S11: asRNAs in genes encoding transposases, highlighted asRNAs overlap the 5′ end of transposases, Table S12: Ribosome associated asRNAs, Table S13: aTSSs comparison between *H. salinarum* and *H. volcanii,* Table S14: Conserved asRNAs differentially expressed in *H. volcanii* during oxidative stress
