# Supplementary material for: The Primary Antisense Transcriptome of Halobacterium salinarum NRC-1
Source: Genes (Basel). 2019 Apr 5;10(4):280. doi: 10.3390/genes10040280 (PMC6523106; doi:10.3390/genes10040280)
Supplement: Supplementary file 1 [file genes-10-00280-s001.zip › Supplementary_figures.pdf]

Article

The primary antisense transcriptome of *Halobacterium salinarum* NRC-1

João Paulo Pereira de Almeida [1,$], Ricardo Z. N. Vêncio [2,$], Alan P. R. Lorenzetti [1], Felipe ten-Caten [1], José Vicente Gomes-Filho [1] and Tie Koide [1,*]

[1] Department of Biochemistry and Immunology, Ribeirão Preto Medical School, Universidade de São Paulo, Brazil
[2] Department of Computation and Mathematics, Faculdade de Filosofia, Ciências e Letras de Ribeirão Preto, Universidade de São Paulo, Brazil
$ authors contributed equally
* Correspondence: tkoide@fmrp.usp.br; Tel.: +55-16-3315-3107

Supplemental Figures

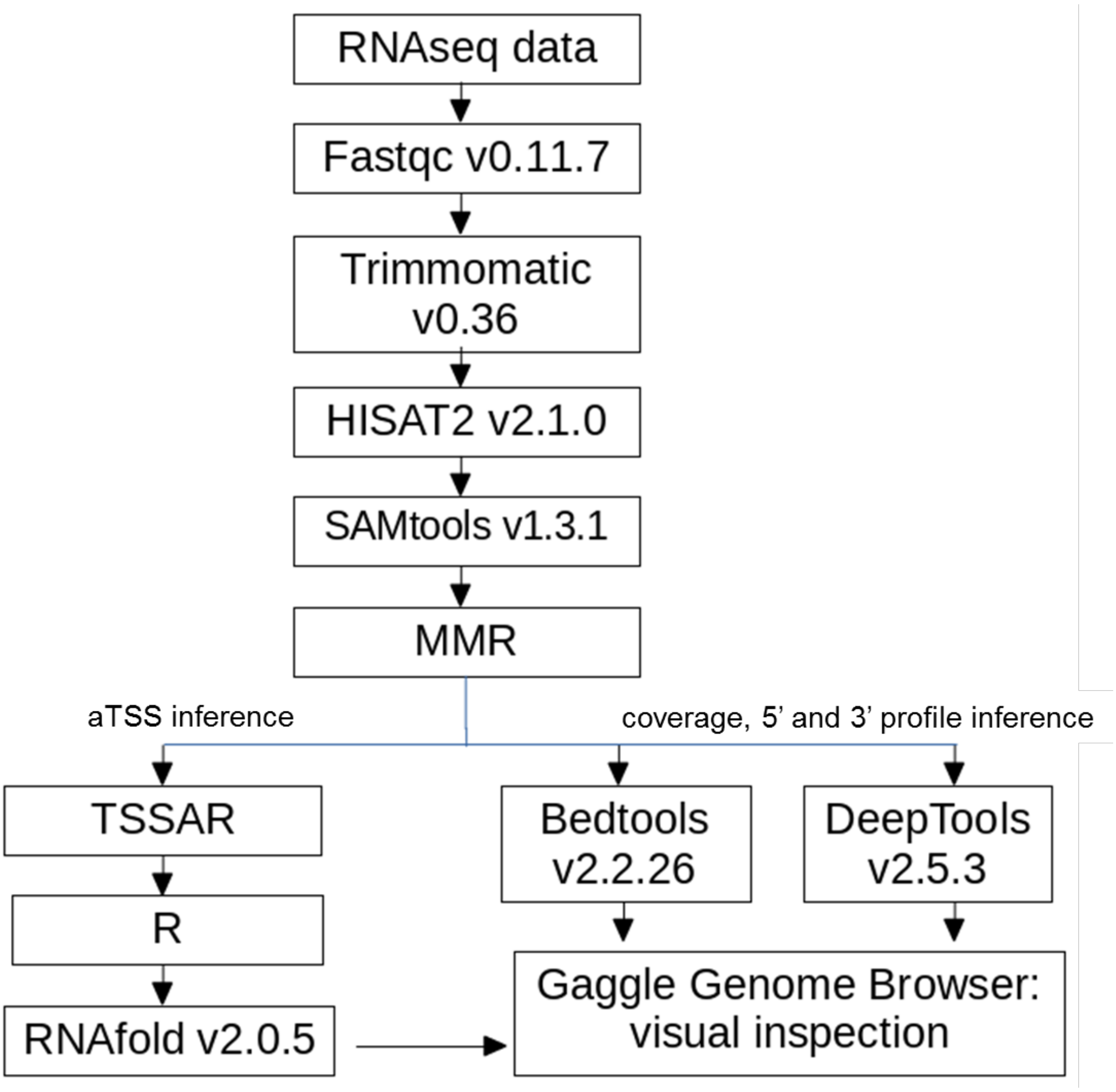

Figure S1. Pipeline for asRNA annotation.

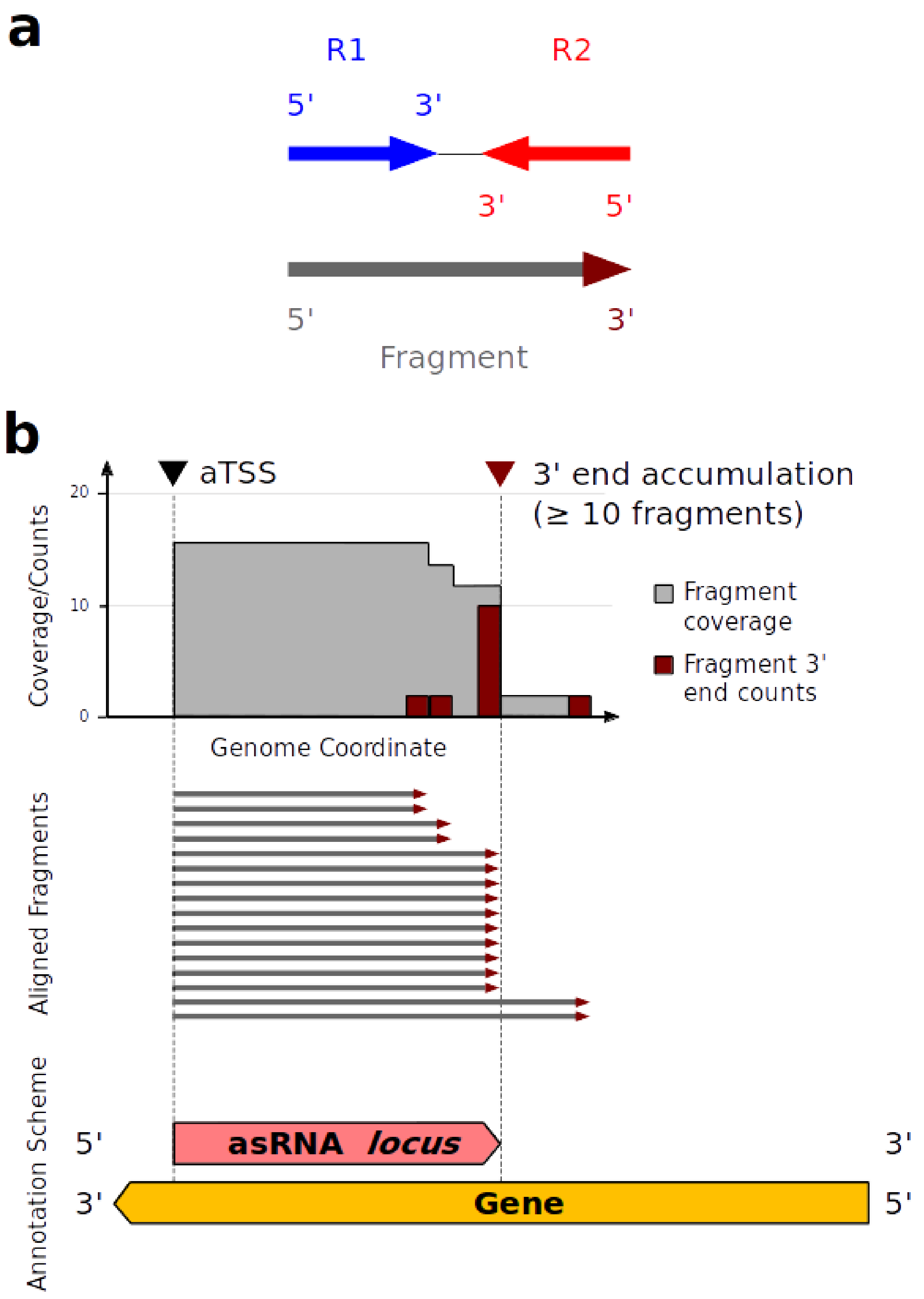

Figure S2. asRNA *loci* inference approach. (a) Reads aligned as a pair (R1-R2; blue and red arrows, respectively) are a representation of an existent RNA fragment (grey line with brown arrowhead), since our RNA library preparation protocol ligates adapters directly to 5′ and 3′ ends of RNA molecules. Furthermore, we can extrapolate that orphan R2 reads aligned using single-end mode also have implicit information about the ending position of a fragment. (b) In our approach, the minimum size of an asRNA locus (pink arrow) can be inferred by mapping its beginning (black triangle), given by an aTSS position, and its minimum ending position (brown triangle), given by the accumulation of at least 10 observations of fragment 3′ ends.

(a)

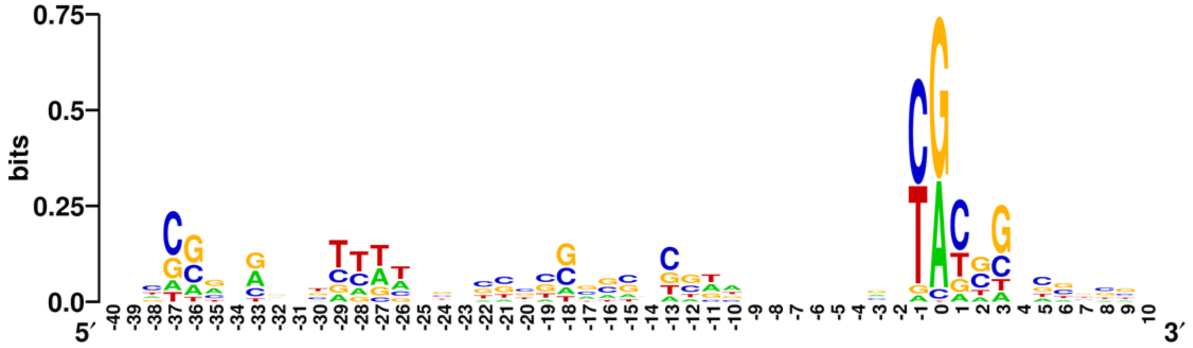

(b)

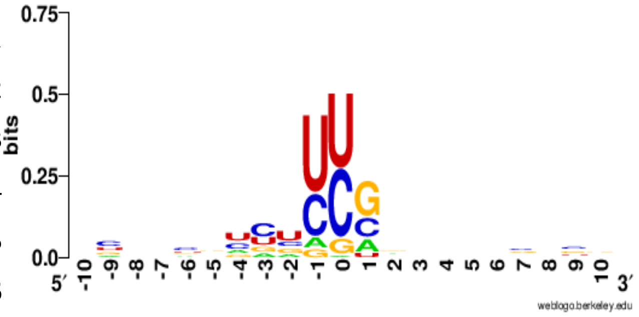

Figure S3. asRNA properties. (a) Promoter analysis: frequency of nucleotides 40 nt upstream and 10 nt downstream of aTSS (position 0). (b) Frequency of nucleotides at the 3′ end of asRNAs. Position 0 is the last nucleotide.

(a)

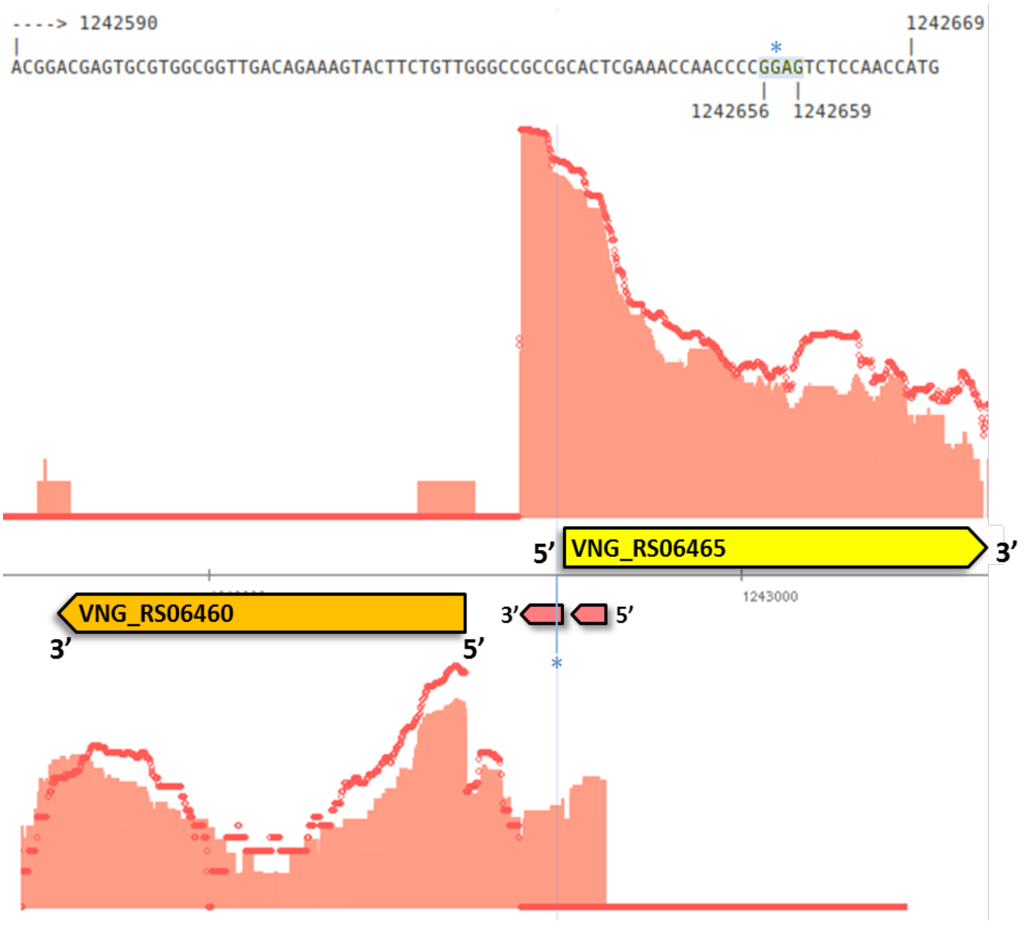

----> 1242590
1242669
ACGGACGAGTGCGTGGCGGTTGACAGAAAGTACTTCTGTTGGGCCGCCGCACTCGAAACCAACCCCGGAGTCTCCAACCATG
1242656
1242659
5'
VNG_RS06465
3'
1243000
VNG_RS06460
3'
5'
3'
5'

(b)

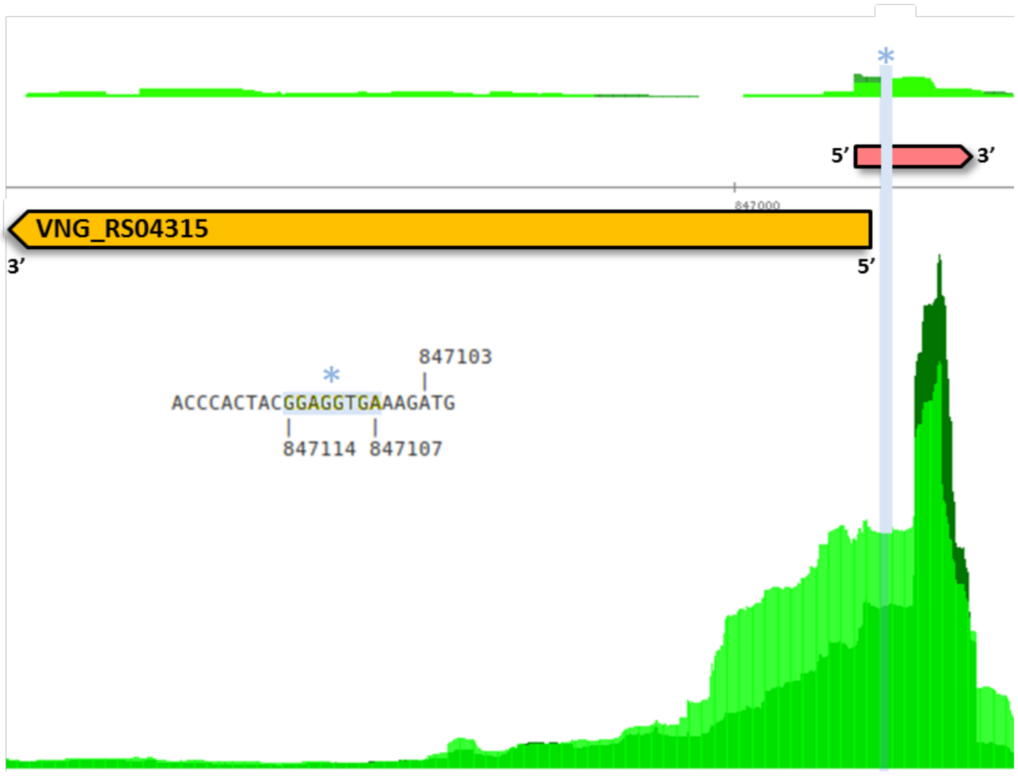

5'
3'
VNG_RS04315
3'
5'
847103
ACCCACTACGGAGGTGAAAGATG
847114
847107

Figure S4. Examples of putative RBS occlusion by an asRNA. (a) *cdcH*, encoding an AAA-type ATPase (VNG_RS06465). VNG_RS06465 *locus* (yellow arrow) is in forward strand (5′→3′ left to right), neighbor gene VNG_RS06460 (orange arrow) is in reverse strand (5′→3′ right to left). Aligned reads coverage along genomic coordinates for TEX+ libraries at exponential and stationary phases are shown in light red (solid) and red (dots), respectively (log_2 counts jointly normalized and arbitrarily scaled). VNG_as06465_925 asRNA (pink arrow) encompasses Shine-Dalgarno-like signature (* light blue highlight). (b) *rpl1* encoding the 50S ribosomal protein L1. VNG_RS04315 *locus* (orange arrow) in reverse strand (5′→3′ right to left). dRNA-seq read coverage signal is shown in dark and light green for TEX+ and TEX- libraries, respectively. VNG_as04315_654 asRNA (pink arrow) encompasses Shine-Dalgarno-like signature (* light blue highlight in genome coordinates and zoomed in sequence).

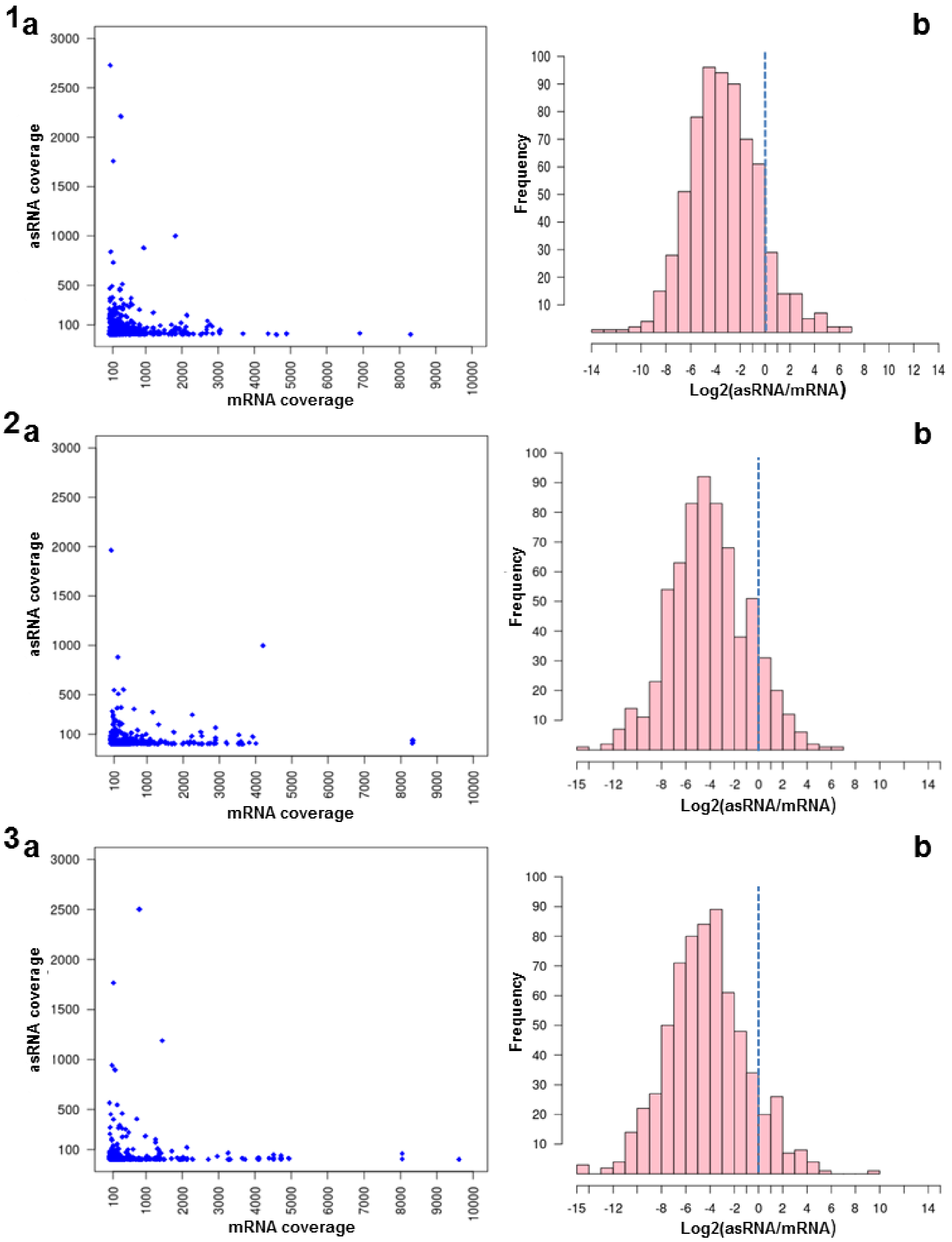

Figure S5. Coverage of asRNAs relative to mRNAs on the opposite strand. (a) Coverage of mRNAs (x-axis) and coverage of asRNAs (y-axis). (b) Histogram of log_2 fold change of asRNA coverage relative to mRNA on the opposite strand; vertical dotted lines mark 1:1 expression levels. (1) stationary phase -17 h, (2) beginning of exponential phase -37 h, (3) gas vesicles release phase – 86 h.

(a)

	Log2FC			
aTSS_2145	3.6	1.2	VNG_RS12020	type IV pilin
aTSS_617	3.6	1.3	VNG_RS04115	hypothetical protein
aTSS_1347	2.7	3.1	VNG_RS09530	argininosuccinate lyase
daTSS_80	2.3	2.3	VNG_OE5069A1	conserved hypothetical protein
● aTSS_1949	2	-1	VNG_OE5075F	conserved hypothetical protein
● aTSS_1426	1.8	3.3	VNG_OE4600F	hypothetical protein
daTSS_68	1.2	2.6	VNG_RS12750	hypothetical protein
aTSS_49	1.2	-1.3	VNG_RS00175	cell division control protein Cdc6
● aTSS_810	-1	1.4	VNG_RS05605	hypothetical protein
daTSS_3	-1.4	2.7	VNG_RS00140	type II toxin-antitoxin system HicA family toxin
aTSS_390	-2	-1.5	VNG_RS02565	oxidoreductase
aTSS_1736	-2	-1.3	VNG_RS12765	site-specific integrase
daTSS_82	-2.1	1.9	VNG_RS13545	hypothetical protein
aTSS_84	-2.2	-2.2	VNG_RS00235	hypothetical protein
aTSS_32	-2.4	-1.1	VNG_RS00115	hypothetical protein
● aTSS_742	-2.6	1.2	VNG_RS05060	class IV adenylate cyclase
aTSS_184	-2.7	1.2	VNG_RS00870	integrase
aTSS_231	-2.8	1	VNG_RS01200	ATP-dependent protease LonB
aTSS_20	-2.8	1.5	VNG_RS00105	transposase
aTSS_1884	-2.9	-1.4	VNG_RS11055	transferase
aTSS_701	-2.9	-1.6	VNG_RS04790	cell division control protein Cdc6
aTSS_1735	-3	-1.3	VNG_RS12765	site-specific integrase
aTSS_908	-3.1	1.8	VNG_RS06345	halocyanin
● aTSS_1321	-3.6	-3.2	VNG_RS09335	ribonucleotide-diphosphate reductase subunit beta
● aTSS_539	-3.6	1.7	VNG_RS03715	hypothetical protein
aTSS_33	-5.9	-1.1	VNG_RS00115	hypothetical protein

(b)

Log2FC

asRNA	log2FC asRNA	log2FC mRNA	mRNA
aTSS_1484	5.8	4.3	VNG_RS10400
aTSS_999	4.9	-1.2	VNG_RS07090
aTSS_203	4.2	1.3	VNG_RS00885
aTSS_62	4.2	1.9	VNG_RS13210
● aTSS_297	4.1	2.6	VNG_RS01695
aTSS_2057	3.8	2.7	VNG_RS13595
aTSS_571	3.8	1.7	VNG_RS03850
aTSS_573	3.8	2.4	VNG_RS03865
aTSS_919	3.3	1.3	VNG_RS06405
aTSS_122	3	1.3	VNG_RS00420
aTSS_1898	3	1.4	VNG_RS11080
aTSS_1762	3	2	VNG_RS12815
● aTSS_78	2.9	2.4	VNG_RS00215
aTSS_1763	2.7	2	VNG_RS12815
● aTSS_826	2.5	2.9	VNG_RS05750
aTSS_1485	2.5	4.3	VNG_RS10400
aTSS_86	2.4	1.4	VNG_RS00240
● aTSS_2050	2.4	2.9	VNG_RS13590
aTSS_187	2.3	2	VNG_RS13265
aTSS_362	2.3	1.1	VNG_RS02300
aTSS_1731	2.2	2	VNG_RS12755
aTSS_922	2.1	1.8	VNG_RS13355
aTSS_65	2.1	1.9	VNG_RS13210
aTSS_1732	1.9	2	VNG_RS12755
aTSS_1923	1.8	1.4	VNG_RS11165
● aTSS_2013	1.8	1.9	VNG_RS13565
● aTSS_1915	1.7	1.6	VNG_RS11110
● aTSS_373	1.2	1.1	VNG_RS02420
aTSS_363	-2.3	1.1	VNG_RS02300
aTSS_2056	-3.3	1.2	VNG_RS11690

transposase
CTP synthetase
hypothetical protein
hypothetical protein
sodium:proton antiporter
hypothetical protein
integrase
hypothetical protein
hypothetical protein
restriction endonuclease subunit S
hypothetical protein
pyridoxamine 5'-phosphate oxidase family
hypothetical protein
hypothetical protein
pyridoxamine 5'-phosphate oxidase family
hypothetical protein
hypothetical protein
transposase
hypothetical protein
hypothetical protein
DUF4352 domain-containing protein
cytochrome b
hypothetical protein
ATP-binding protein
hypothetical protein
hypothetical protein
chromosome segregation protein SMC
hypothetical protein
hypothetical protein
hypothetical protein
cytochrome b
IS4 family transposase ISH30

Figure S6. Differential expression of asRNAs and mRNAs on the opposite strand. Pairs asRNA/mRNA that presented either positive or negative correlation are shown. Up-regulated genes have its log fold change (log_2FC) inside red cells and down-regulated genes inside green cells. (a) log_2FC for 37 h/17 h (b) log_2FC for 86 h/37 h. Orange dots highlight asRNAs overlapping the 5′ UTR of the mRNA.

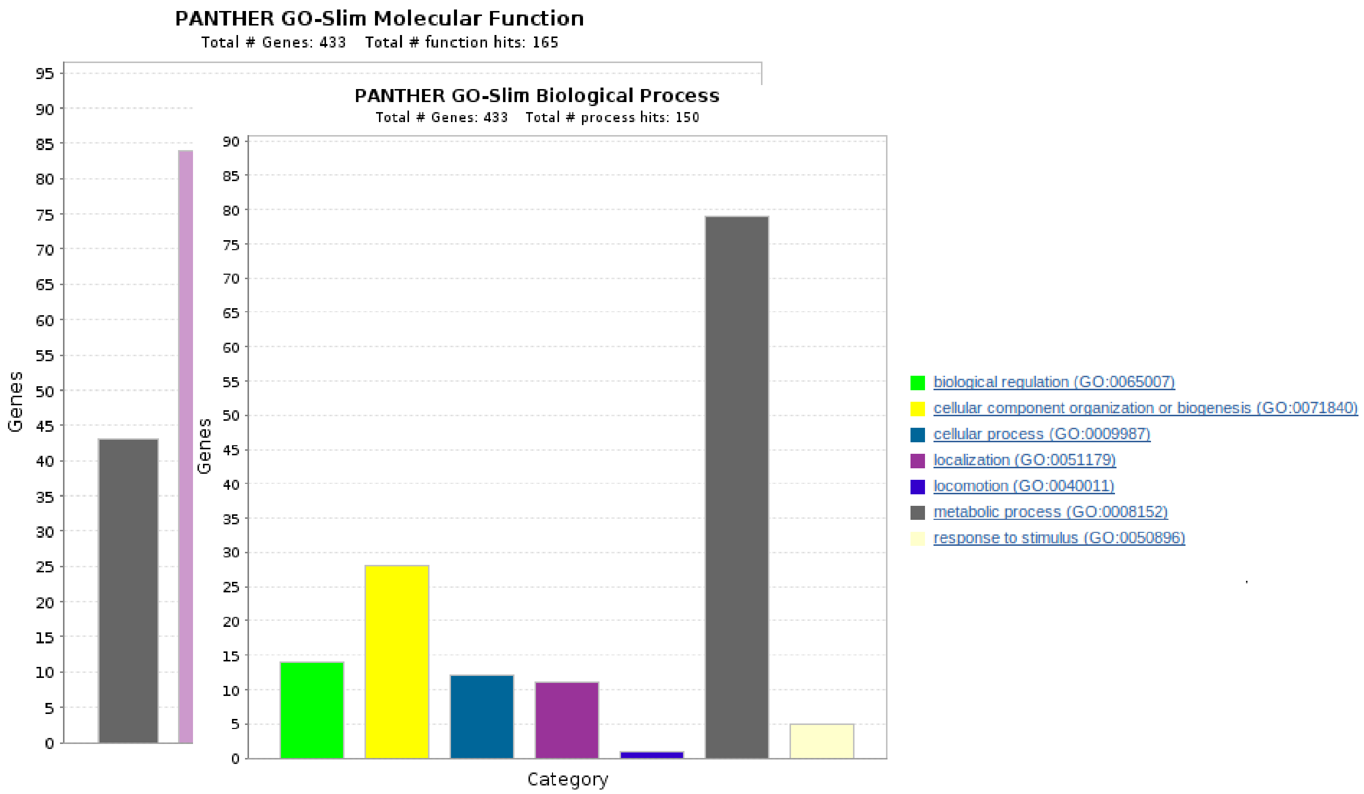
PANTHER GO-Slim Molecular Function
Total # Genes: 433 Total # function hits: 165
Genes
PANTHER GO-Slim Biological Process
Total # Genes: 433 Total # process hits: 150
Genes
Category
biological regulation (GO:0065007)
cellular component organization or biogenesis (GO:0071840)
cellular process (GO:0009987)
localization (GO:0051179)
locomotion (GO:0040011)
metabolic process (GO:0008152)
response to stimulus (GO:0050896)

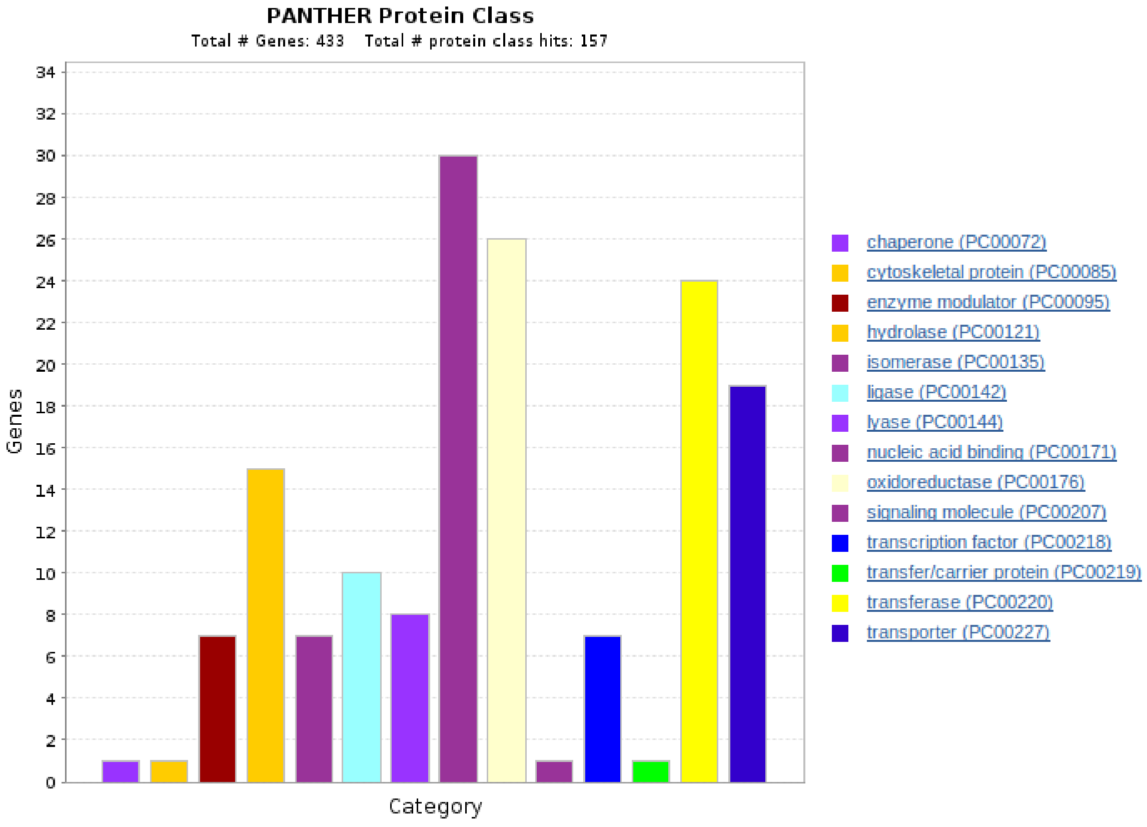

Figure S7. Functional categorization of genes presenting asRNAs according to Gene Ontology (GO). PANTHER tool was able to classify 433 genes out of the 613 submitted. Panels show molecular function, biological processes and protein classes, respectively.

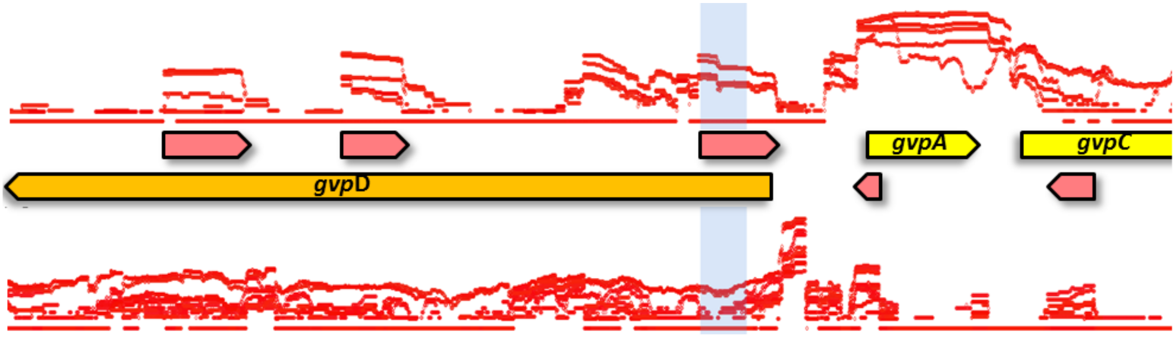

Figure S8. asRNAs in *gvp* genes. Upper panel shows RNA-seq log_2 counts for forward strand, and lower panel shows signal for the reverse strand. All sequenced libraries are shown simultaneously and normalized (profiles as red dots). Genes *gvpA* and *gvpC* in forward strand (yellow arrows) and *gvpD* in reverse strand (orange arrow). Annotated asRNAs are represented by pink arrows. asRNAs related to *gvp* genes showed mean expression levels lower than their cognate genes. Region corresponding to the probe used for asRNA detection by Krüger and Pfeifer (1996) is highlighted in light blue.

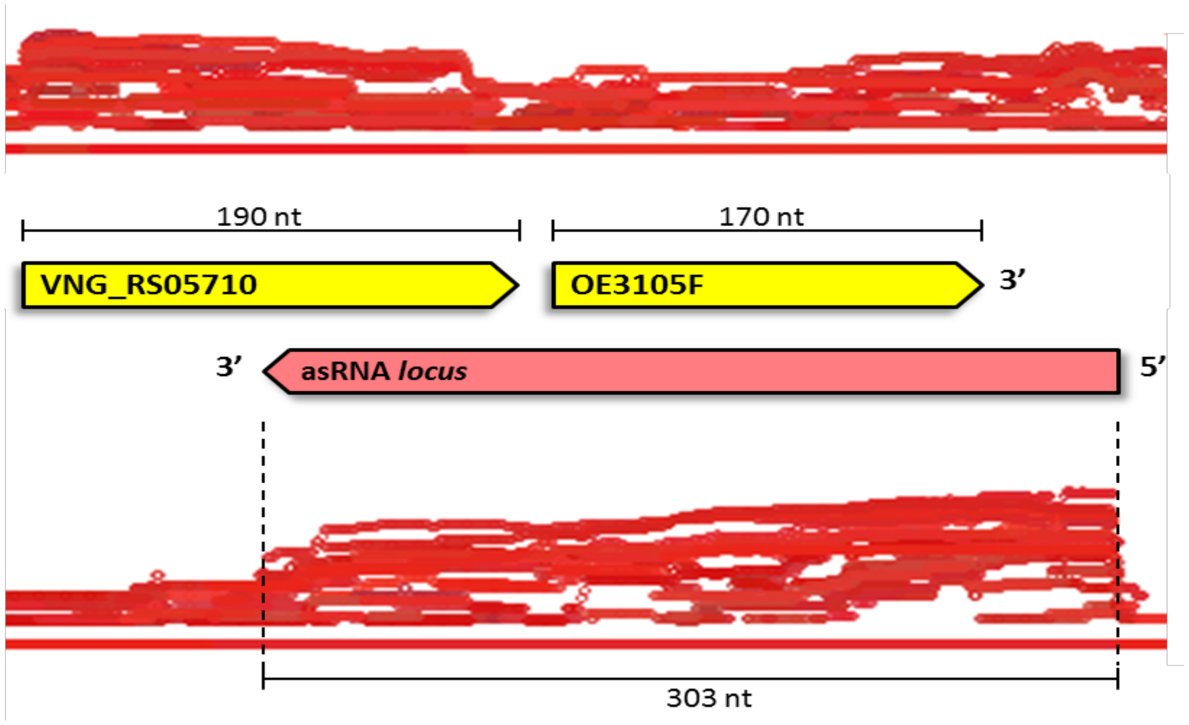

Figure S9. asRNAs in *brb* and *brz* genes. Upper panel shows RNA-seq log_2 counts for forward strand, and lower panel shows signal for the reverse strand. All sequenced libraries are shown simultaneously and normalized (profiles as red dots). *brz* (VNG_RS05710) and *brb* (OE3105F) are on the forward strand (yellow arrows) and annotated asRNA (VNG_da3105F_36) on the reverse strand (pink arrow).

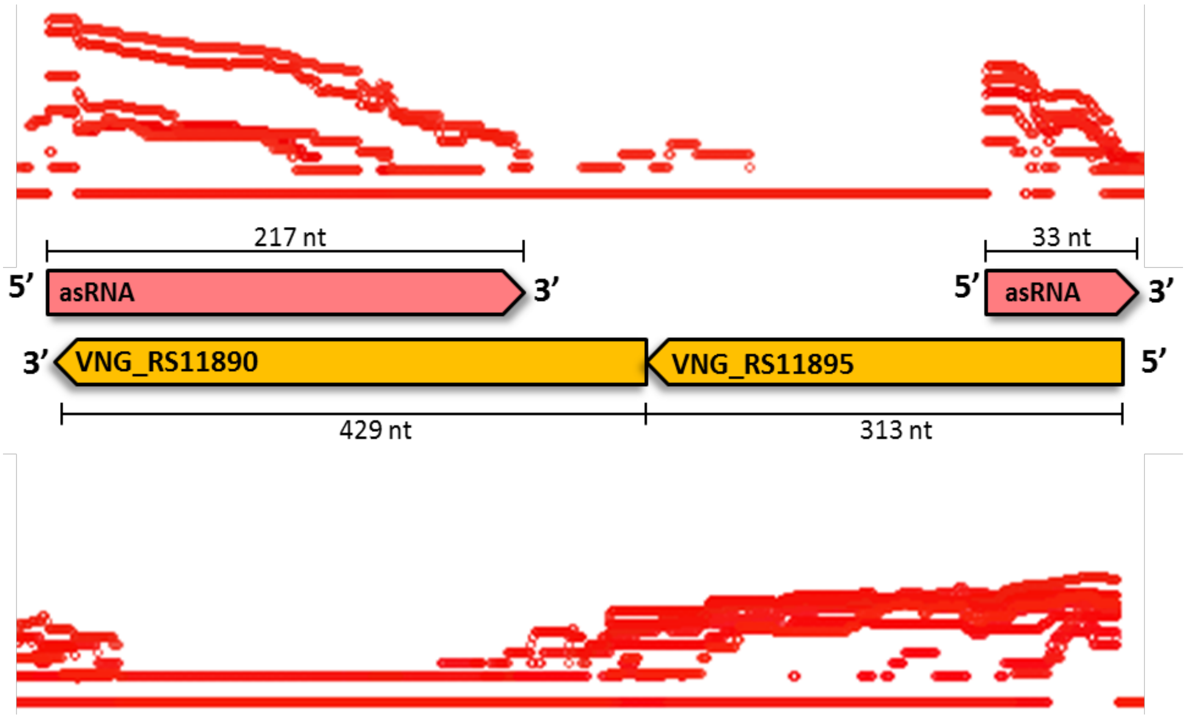

Figure S10. asRNAs in type II TA system. Upper panel shows RNA-seq $\log_2$ counts for forward strand, and lower panel shows signal for the reverse strand. All sequenced libraries are shown simultaneously and normalized (profiles as red dots). Genes VNG_RS11890 (toxin) and VNG_RS11895 (antitoxin) are on the reverse strand (orange arrows) and their respective annotated asRNAs VNG_da11890_90 and VNG_as11895_2118 on the forward strand (pink arrows).

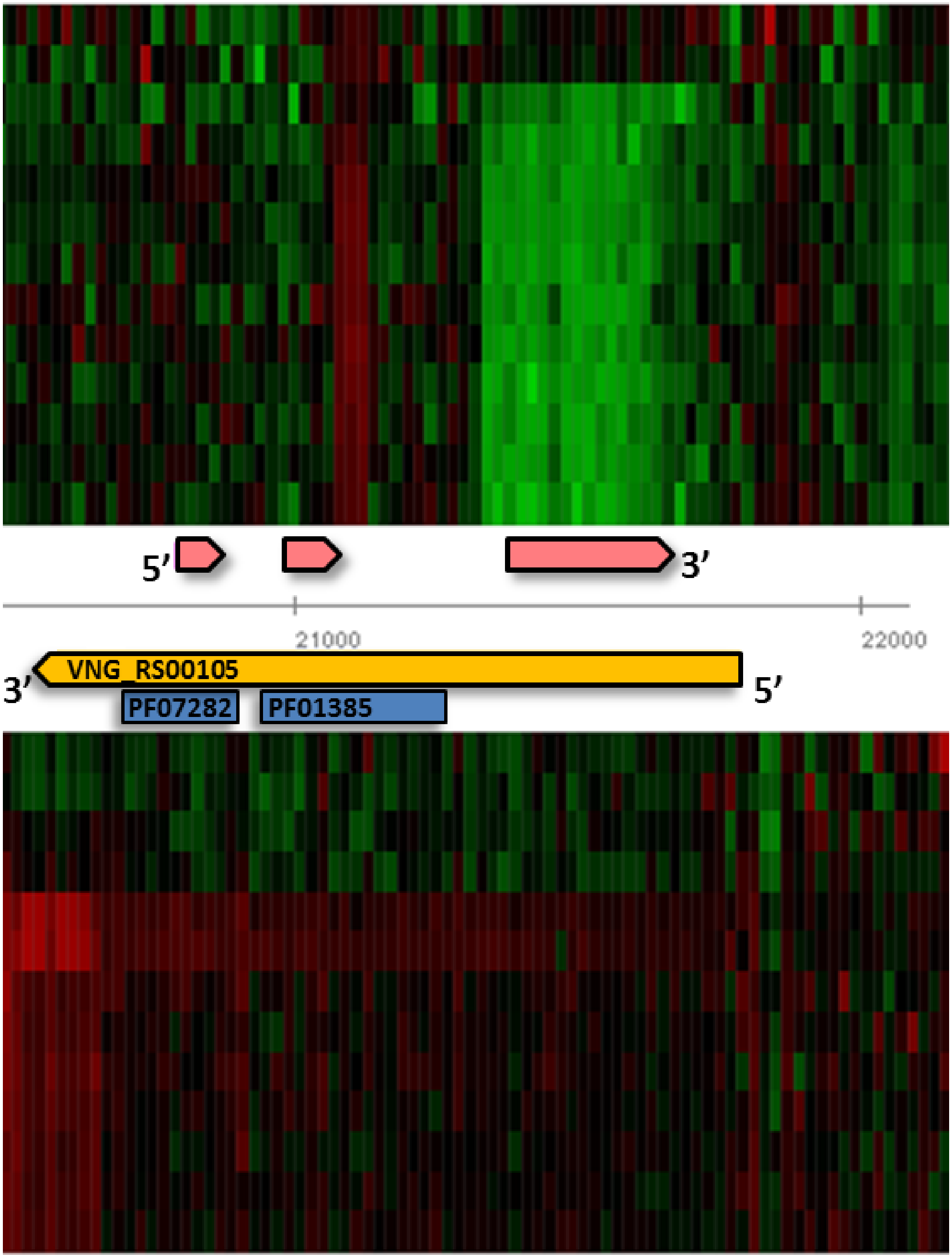

Figure S11. asRNAs antisense to a transposase differentially expressed along the growth curve. VNG_RS00105 (orange arrow) encodes a transposase on the reverse strand (5′→ 3′ right to left), PFAM domains are shown in blue. asRNAs are shown in pink arrows on forward strand (5′→ 3′ left to right). Upper and lower panels show tiling array expression data over a growth curve (Koide *et al.*, 2009) for forward and reverse strand, respectively. Each line represents one point in the growth curve. Heat map is color coded: green shows down-regulation and red up-regulation relative to reference condition.

(**a**)

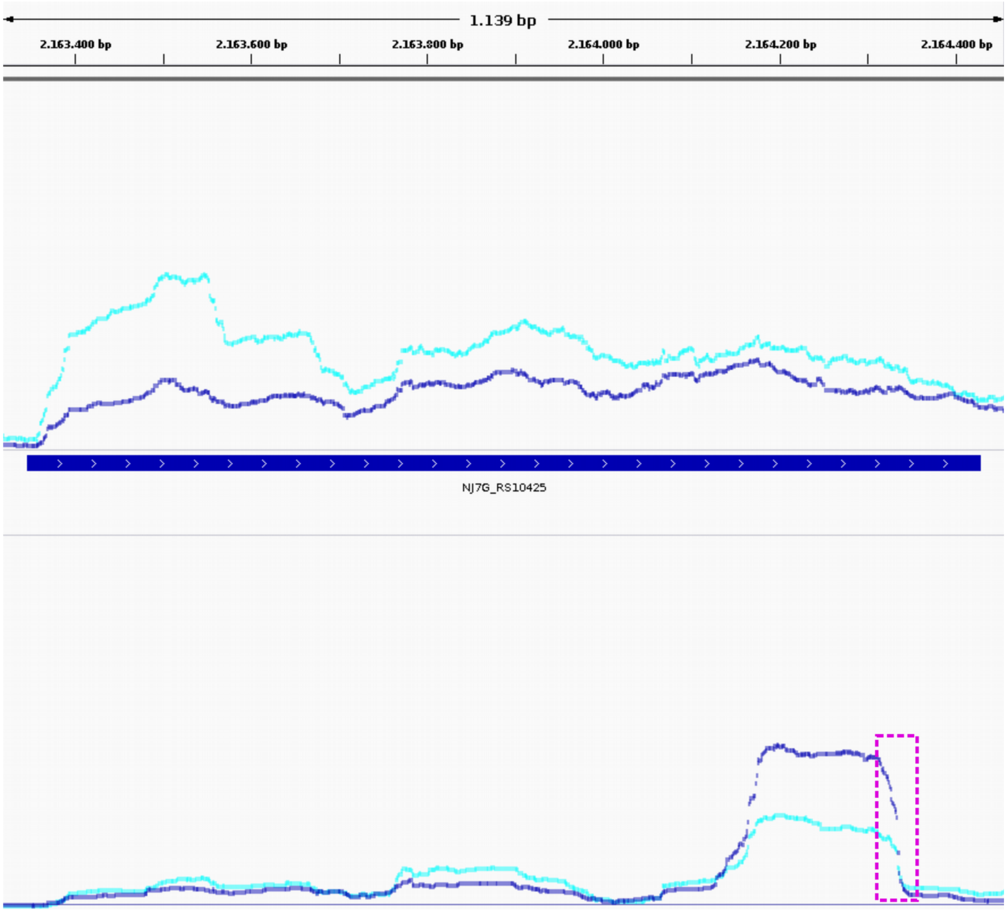

(**b**)

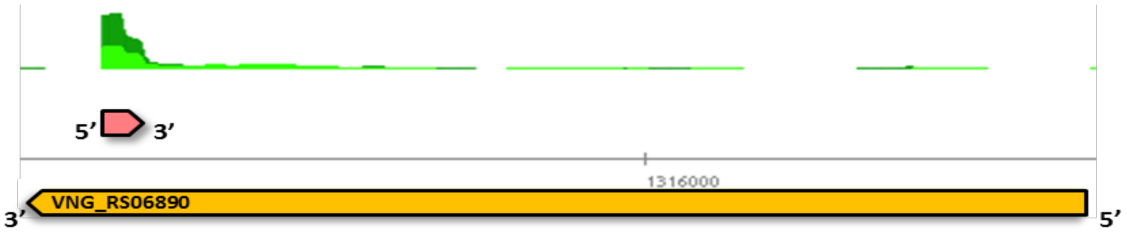

Figure S12. Differential expression of putative asRNAs antisense to the *nirH* gene in *Natrinema* sp. J7-2. (a) Genomic region showing normalized read coverage for low (15% NaCl; light blue) and high (30% NaCl, dark blue) salt concentrations, respectively. The y-axes range from 0 to 500 counts. CDS in *Natrinema* sp. J7-2 (blue rectangle) is shown at forward strand (5'→3' left to right). Signals above and below the blue rectangle are for forward and reverse strands, respectively. A highlight box (magenta) indicates the approximate asRNA *locus* position identified in *H. salinarum.* (b) *H. salinarum* dRNA-seq read coverage signal is shown in dark and light green for TEX+ and TEX- libraries, respectively (5'→3' right to left). AsRNA *locus* position is highlighted (magenta). Coverage signal above the axis is for forward strand.

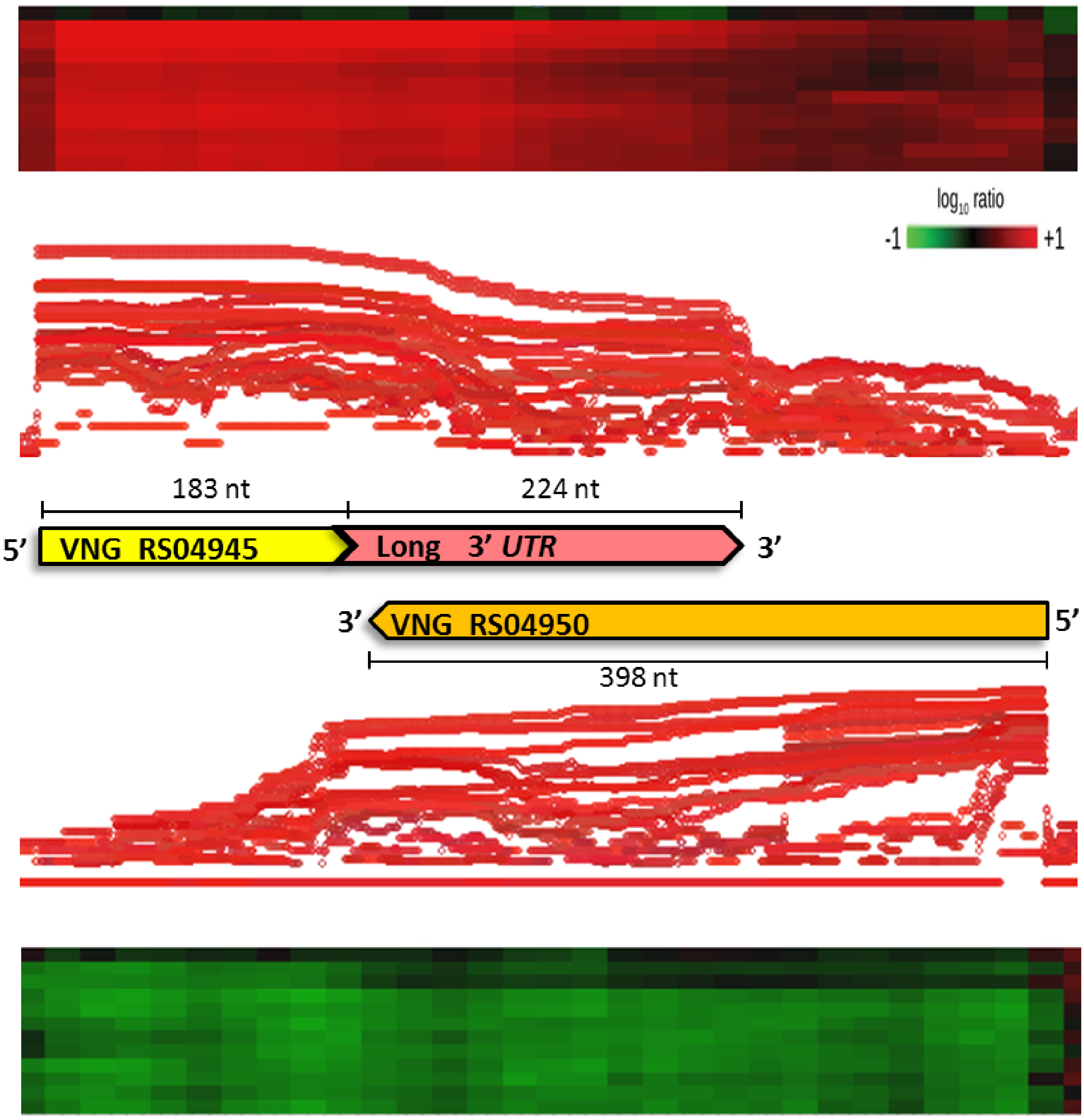

Figure S13. Long 3′ UTR of a coding sequence acting as an asRNA. Pink arrow indicates a possible long 3′ UTR of the gene VNG_RS04945 (yellow arrow, annotated as hypothetical protein) which could act as an asRNA of the gene VNG_RS04950 (orange arrow, annotated as a subunit of a translation initiation factor). Upper panel shows tilling array expression data for the forward strand and lower panel for the reverse (Koide *et al.* 2009). Red lines show RNA-seq signal ($\log_2$ counts per position).

© 2019 by the authors. Submitted for possible open access publication under the terms and conditions of the Creative Commons Attribution (CC BY) license (http://creativecommons.org/licenses/by/4.0/).
